# Does an online psychological intervention improve self-efficacy and disability in people also receiving Multimodal Manual Therapy for chronic low back pain compared to Multimodal Manual Therapy alone? Design of a randomized controlled trial

**DOI:** 10.1186/s12998-015-0080-9

**Published:** 2015-12-18

**Authors:** M. John Petrozzi, Andrew Leaver, Mairwen K. Jones, Paulo H. Ferreira, Sidney M. Rubinstein, Martin G. Mackey

**Affiliations:** Research Student, Faculty of Health Sciences, The University of Sydney, Sydney, Australia; Discipline of Physiotherapy, Faculty of Health Sciences, The University of Sydney, Sydney, Australia; Discipline of Behavioural and Social Sciences in Health, Faculty of Health Sciences, The University of Sydney, Sydney, Australia; Faculty of Earth and Life Sciences, Institute of Health Sciences, VU University Amsterdam, Amsterdam, The Netherlands

**Keywords:** Chronic LBP (LBP), Self-efficacy, Disability, Multimodal manual therapy (MMT), Chiropractic, Physiotherapy, Behavioural change, MoodGYM, ICBT

## Abstract

**Background:**

Various interventions are available for the treatment of chronic low back pain (LBP), including Manual Therapy and Cognitive Behavioural Therapy (CBT). The purpose of this study is to evaluate whether the addition of an internet-based CBT program leads to better outcomes in patients who are treated with multimodal manual therapy for chronic LBP.

**Methods/Design:**

A randomized controlled trial comparing a combined intervention, consisting of internet-based CBT utilising MoodGYM plus multimodal manual therapy, to multimodal manual therapy alone for patients with chronic LBP. Multimodal manual therapy will be delivered by experienced chiropractors and physiotherapists. Treatment sessions will consist of a combination of joint and soft tissue mobilisation; spinal manipulation as well as muscle and fascia massage; education and reassurance; and rehabilitative exercise prescription. In total, 108 adult participants will be recruited from multiple chiropractic and physiotherapy private practices in Australia. Participants older than 18 years of age and diagnosed with chronic non-specific LBP will be included in the trial, where chronic LBP is defined as continuous or fluctuating pain for a minimum of three months.

The Keele STarT Back screening tool will be used to screen for potential participants who are in the medium risk category. The primary outcomes are self efficacy and disability measured by the Patient Self-Efficacy Questionnaire (PSEQ) and Roland Morris Disability Questionnaire (RMDQ) respectively. Secondary outcome measures will assess pain, catastrophising, depression, anxiety, stress and work ability. Participants will be randomly allocated into one of two groups. Both groups will receive an upper limit of 12 multimodal manual therapy sessions over a period of 8 weeks. The intervention group will also receive five weeks of MoodGYM covering five modules in total. Assessment will be conducted at pre-treatment, post-treatment 8- and follow-up at 26- and 52 weeks. In addition, a verbal pain measure will be completed by the treating practitioner at time of treatments on an 11-point VAS. The primary data analysis will be by intention to treat using a linear mixed model for each outcome.

**Discussion:**

This paper outlines the design of a randomised controlled trial that investigates the potential benefits of adding a widely available and inexpensive internet-based psychological intervention to standard multimodal manual therapy for the management of chronic low back pain.

**Trial registration:**

ACTRN12615000269538

## Background

Low back pain (LBP) is a highly prevalent health problem and the largest contributor to disability and work absence worldwide [1]. The economic burden of LBP is significant. In Australia, for example, the cost of work days lost due to LBP in 2001 exceeded nine billion dollars [2], which was 1.25% of GDP. It is estimated that 11 % of the population with LBP will develop persisting high intensity pain with high levels of functional disability [3]. Disability in this case refers to functional limitations associated with self-reported back pain [4]. Back pain typically fluctuates over time, with episodes of exacerbation and remission [5]. Much of the research into the management of LBP has focused on the impact that interventions have on improving immediate pain and disability measures over time, and is therefore primarily concerned with the outcome of recovery from a single episode of LBP. Relatively less attention has been given to the secondary prevention of long term moderate level disability, which accounts for the majority of people with LBP still reporting pain or disability more than 12 months after initial onset [6].

Chiropractic and physiotherapy care are popular treatment choices for people with low back pain. Up to 15 % of the Australian population visit a chiropractor every year, most commonly presenting with LBP [7]. Similarly, physiotherapy is frequently used accounting for 17 % of the direct costs of managing back pain in some settings [8]. There is some evidence supporting a range of interventions in the management of chronic LBP which chiropractors and physiotherapists typically perform, including manual therapies, exercise and education [9]. Most of this evidence relates to the role of these interventions in providing short-term improvements in symptoms and function [10]. Manual therapies are often provided as a package of multimodal care that also includes advice, education and exercise [11], that is targeted towards an individual patient’s presentation and determined by the judgment of the practitioner. There is limited evidence about the effectiveness of multimodal manual therapy (MMT) treatments for chronic back pain.

Another option for treating chronic LBP is cognitive behavioural therapy (CBT) [12]. The aim of CBT is to identify, challenge and reframe maladaptive beliefs and behaviours that are associated with pain and disability in people with chronic LBP. This is achieved through pain education, goal setting and learning effective problem solving and pain management strategies. These strategies include activity pacing, daily planning, and confronting avoidance behaviours with the aim of improving mood, resilience and self-efficacy. This is important because low self-efficacy has been shown to predict long term disability [13, 14] and may be an important contributor to the development of disability in people with chronic pain [15]. CBT has been shown to improve measures of self-efficacy in people with chronic LBP [15, 16]. It is likely there may also be a role for CBT in the prevention of long term disability in future new episodes of back pain, by improving a person's resilience, problem solving capacity and coping strategies.

CBT programs, whilst traditionally delivered in a face-to-face format can also be effectively delivered through an internet platform [17]. Internet based programs have the potential advantage of the intervention being easily and broadly accessible at lower cost compared with face-to-face programs [17]. One such internet-based CBT (ICBT) program is MoodGYM [17–19]. MoodGYM is a psychological intervention developed by the National Institute for Mental Health Research at The Australian National University [20]. The goals of MoodGYM are to help people to identify and overcome general psychological distress by developing good psychological coping skills [19]. MoodGYM has met evidence standards for efficacy and effectiveness criteria set by the Society for Prevention Research (SPR) [21], and it has been concluded that it substantially met the standards of evidence for public dissemination [22]. Although MoodGYM has not been formally evaluated in people with LBP, this type of generic training in psychological resilience might be of benefit in people facing the challenges of chronic LBP.

This study will focus on identifying the additional benefits of combining MMT with ICBT in treatment of chronic LBP. We hypothesise that the combination of these two interventions that address different dimensions of the problem of back pain may have additive beneficial effects. MMT has traditionally been more focused on pain reduction and improving physical aspects of performance and function [23]. The combination of easily accessible, generic psychological treatment programs with traditional MMT has shown to improve patient outcomes [24, 25]. The aim of this study is, therefore, to determine whether the addition of an ICBT intervention leads to better outcomes, that is, increased self-efficacy and a lower level of long term disability in those receiving MMT compared to those receiving MMT alone.

## Methods

### Trial design

**Fig. 1:**
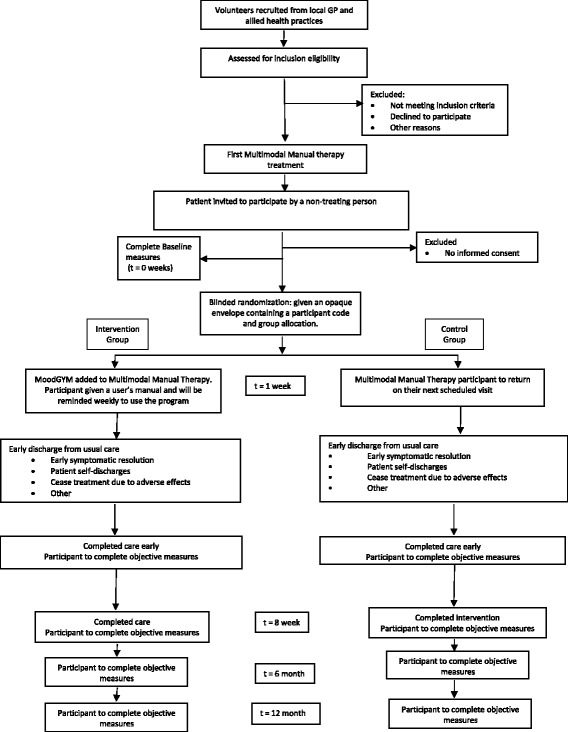
Flow of participants

This will be a randomised controlled trial with two intervention arms that will be conducted in chiropractic and physiotherapy clinics in metropolitan Sydney, Australia. Participants will be randomly allocated to a control group (MMT) or intervention group (MMT plus MoodGYM). An equal ratio of participants will be allocated to each arm of the trial. The trial flowchart is depicted in Fig. 1. The trial has been approved by The University of Sydney Human Research Ethics Committee (protocol number 2014/997). All participants will provide written informed consent prior to entering the trial. The trial was registered prospectively with the Australian New Zealand Clinical Trials Registry Number (ACTRN)12615000269538. Recruitment commenced on 30^th^ March 2015.

### Participants

In total, 108 participants with chronic LBP who are at moderate risk of long-term disability will be recruited through advertisements placed in the participating practices and in general medical and allied health practices in metropolitan Sydney, Australia. Consecutive new patients at the participating practices with LBP will be invited to participate. Potential participants will be screened for inclusion by the treating practitioner using the Keele STarT Back Screening Tool [26] and a standardised form during their initial consultation. Those at moderate risk of developing chronic LBP, scoring in the medium risk category on the STarT Back Screening Tool are the focus of this trial. Eligible participants will be provided with written information about the study and invited to participate, and subsequently requested to provide written informed consent.

### Inclusion criteria

Participants will be included in the study if they are: over 18 years of age, have non-specific LBP of more than three months duration, and are classified as medium risk of chronicity according to the 9-item STarT Back Screening Tool [26].

### Exclusion criteria

Participants will be excluded if they have been diagnosed with serious spinal pathology (fracture, malignancy, infection, inflammatory disorders, canal stenosis or cauda equina syndrome, spinal cord injury), spinal nerve compromise (determined by the presence of two or more corresponding neurological signs such as dermatomal paresthesia or paresthesis, diminished absent deep tendon reflexes), have undergone spinal surgery in the past 12 months, are pregnant, have a compensation claim related to their back condition, are not able to independently complete English language questionnaires, or are not able to independently use a computer.

### Interventions

Participants will be randomly allocated to one of two groups.

**Group 1 Multimodal Manual Therapy (Control):** Participants in this group will receive Multimodal Manual Therapy treatment (MMT) only. MMT refers to treatment that is centred around manual therapies but also employs best practice primary care and is supported by therapeutic exercises. The manual therapy component of MMT may involve high-velocity, low-amplitude thrust manipulation, non-thrust joint mobilization, or soft tissue massage. The high-velocity, low-amplitude thrust techniques will involve a carefully directed, gentle but quick thrust through the mechanical plane of the spine or pelvic joints that may or may not be accompanied by an audible ‘pop’ or cavitation sound [27]. These techniques may be performed with the therapist’s hands or with the assistance of a device such as a drop-piece table. The non-thrust mobilization techniques will involve passive oscillatory movements of the spine or pelvic joints through normal ranges of motion. Soft tissue massage techniques will be directed at the muscles of the back and pelvis and perfomed with the therapist’s hands or a vibrating massage device. All participants will receive components of best practice primary care that involves reassurance, advice about symptom management and encouragement to remain active [9, 28, 29]. MMT may be supported by exercises that may include general physical conditioning, or specific exercise programs that address; symptom management, or mobility, strength or motor control impairments. Other treatment modalities that are used by chiropractors and physiotherapists that are not endorsed by practice guidlines (e.g. therapeutic ultrasound, transcutaneous electrical nerve simulation, heat therapy, etc) will not be included in the definition of MMT.

All participants in the MMT group will receive manual therapy. The selection of other supportive treatment modalities will be determined by the chiropractor or physiotherapist according to their clinical judgment. Treatments will either be performed by a registered chiropractor or physiotherapist who has more than 5 years of clinical experience. Participants will receive up to 12 treatments over a period of 8 weeks. The practitioner may elect to use fewer treatments in cases where the participant has experienced significant improvement or has developed adverse effects that warrant stopping care. The definition of an adverse effect (event) is either: 1) a new related complaint which was not present at baseline or previous visit, or 2) a worsening of the presenting complaint [30]. Adverse events will be measured using a similar questionnaire as in the side effects of chiropractic treatment study by Leboeuf-Yde et al. [31] and will be carried out at the 8 week follow up interval.

**Group 2 MoodGym combined with Multimodal Manual Therapy (Intervention):** Participants in this group will receive the same MMT as Group 1; however, this group will also complete the MoodGYM program [22]. Participants will complete one module of the MoodGYM program each week over the first five weeks of the trial. Participants in the MoodGYM group will set up a username and password at the time of enrolment and will be given an instruction manual for their five-week online MoodGYM program.

Participants will also receive a weekly telephone reminder, to enhance adherence with the MoodGYM program and avoid attrition. Attrition has been identified as an issue in similar studies and higher completion rates were achieved with occasional telephone or email contact [20]. No additional counseling or treatment advice will be provided with the reminder telephone calls. Participants who report distress as a result of using MoodGym will be offered referral to a clinical psychologist.

Participants in both groups will be given a follow-up appointment schedule for their 8-week course of MMT. The MMT will be provided by a chiropractor or a physiotherapist using techniques listed above which are inaccordance with International Clinical Practice Guidelines for chronic LBP [9, 28, 29].

#### Randomisation and blinding

An independent person not involved in participant recruitment, treatment delivery, data collection or analysis will use a computer software formula to generate a random number sequence to produce an even number of participants for group allocation prior to commencement of the trial. Group allocation for each participant will be sealed in a single consecutively numbered, opaque envelope.

Randomisation will occur following the initial manual therapy consultation. The allocation envelope will be opened by a research assistant who is not involved in the participant’s MMT. The research assistant will then provide participants with instructions about completing their interventions. Participants in the control group will be provided with their appointment schedule for future treatments. Participants in the intervention group will be provided with their treatment appointment schedule and provided with a user manual for the use of MoodGym. It is important to note that the participants in this trial will not be given any assistance above and beyond what is already available for regular internet users.

It is not possible to blind the participants to the nature of the intervention that they will receive. However, participants in the control group will receive limited information about the other intervention. The participants in both groups are unaware of the exact online psychological intervention being used in the trial as MoodGYM is not mentioned at any time during advertising or participant recruitment. After randomization, only participants in the intervention group will be told about MoodGYM. Additionally an Expectancy of Change Questionnaire will be completed by both groups at the time of enrolment to measure their expectations and how they rate the credibility of the intervention. The treating practitioner will be blinded to group allocation: Participants will be instructed to not reveal their group allocation or discuss the MoodGym program with the treating practitioner. Outcome data will be collected by a researcher who is blinded to treatment allocation.

### Data collection and outcome measures

Baseline questionnaires will be administered at the initial consultation by the treating practitioner. Baseline data will include demographic and clinical characteristics including participant gender, age, work status, functional impairment, treatment history and medical history, pain rating, self-efficacy, catastrophizing, stress anxiety and depression scores and work ability. Outcome data will be collected through questionnaires at 8-, 26- and 52 weeks follow-up. In addition, a verbal pain measure will be taken by the treating practitioner at time of treatments on an 11-point VAS.

#### Primary outcomes

The primary outcomes are between group differences in self-efficacy, measured using the Pain Self Efficacy Questionnaire (PSEQ) and disability measured using the Roland Morris Disability Questionnaire (RMDQ) at the conclusion of treatment (8 weeks), 6- and 12-month follow up. The PSEQ is a 10-item questionnaire that measures self-efficacy on a scale from 0 to 60, where a higher score reflects higher self-efficacy [32]. A higher self-efficacy rating correlates with a patient’s strong belief that they can manage to execute an activity. This scale has strong psychometric qualities [33] with alpha values reported as 0.93 [32]. The RMDQ is a 24 item questionnaire that scores an individual’s level of disability from 0 to 24, ranging from no disability (0) to severe disability (24) [34]. The responsiveness rate of the RMDQ has been calculated to be between 0.76–0.78 in some studies [35].

#### Secondary outcomes

Secondary outcomes include Pain Catastrophizing Scale (PCS) [36], Patient Specific Functional Scale (PSFS) [37], Depression Anxiety and Stress Scale (DASS21) [38], Pain Numerical Rating Scale (PNRS) [39] and Work Ability, using the single item Work Ability Index (WAI) score [40] at baseline, 8 weeks, 6 months and 12 months.

- Pain Catastrophizing Scale (PCS) measures a person’s tendency to excessively focus on the sensations of pain (rumination), the threat of pain (magnification), and to subordinate to a feeling that they have no control over the intensity of pain (helplessness) [41]. Three subscale scores describe the levels of rumination, magnification and helplessness. This scale has high internal reliability [42] (coefficient alphas: total PCS = 0.87, rumination = 0.87, magnification = 0.66, and helplessness = 0.78 [36].
- Using the Patient Specific Functional Scale (PSFS) participants are asked to identify three important activities that they are unable to do, or have difficulty doing because of their LBP. Each is scored between 0 and 10 on a Likert scale. The PSFS tool is used to quantify any activity limitations, as well as measuring any functional improvements or outcomes over time [37].
- The DASS21 is a validated 21 question tool that measures depression, anxiety and stress in adults [38]. It has been developed as a short form of the DASS, which is a self-reported scale with three subscales; Depression (DASS-D), Anxiety (DASS-A) and Stress (DASS-S). The psychometric properties of this scale have been noted as 0.94 (DASS-D), 0.87 (DASS-A) and 0.91 (DASS-S) [43].
- The Pain Numeric Rating Scale measures a participant’s pain intensity on an 11 point scale (0-10). Participants are asked about their present pain, as well as pain intensity over the preceding week for: usual, best and worst pain levels. The scale is a valid and sensitive tool [39].
- This trial uses a modified version of the full 7 sectioned WAI that has been assessed for validity and reliability by the European Network for Workplace Health Promotion (ENWHP) and National WAI Network [40]. The question is: “Rate your work ability now, compared to your life time best, on a scale of 1 to 10 (1 is worst, 10 is best)”. A higher score indicates the person’s good self-reported work ability, and conversely, a lower value indicates indicated a jeopardised work ability rating.

#### Statistical methods

The primary data analyses will be conducted using linear mixed models to test for between group differences in post-treatment PSEQ and RMDQ. A covariance structure will be selected that provides the best fit and then will be included as a random factor to adjust for a possible cluster effect of site. A group by time interaction will be included to allow least significant difference (LSD) contrasts to be obtained between groups at each time point. Because linear mixed models allow for missing data points, no imputation of missing data values will be required. Analysis will be by intention-to-treat with the statistician blinded to patient group allocation.

In secondary analyses, analysis of covariance will be used to test whether regression to the mean has occurred, that is whether the patient’s response is related to baseline values. In this, separate analyses will be conducted to determine the effects of treatment at −8, −26 and −52 weeks.

#### Sample size calculations

With an estimated moderate post-treatment between-group effect size (Glass’s delta) of 0.60 SD, a sample size of 46 per group would be required to show statistical significance (power of 80 %, alpha = 0.05) using a LSD post hoc test in a linear mixed model (equivalent to an independent samples *t*-test). Allowing for a 15 % dropout, 108 participants in total will be recruited. We are assuming that the potential loss of power as a result of a cluster effect will be balanced by the use of linear mixed models to negate the effects of missing values and the increased power gained by adjusting for between visit correlations.

## Discussion

This paper outlines the design of a randomised controlled trial that investigates the potential benefits of adding a widely available and inexpensive intervention to standard MMT.

Numerous chronic LBP Clinical Practice Guidelines have recommended that patient management for LBP should consist of MMT and CBT [9, 29, 44, 45]. MMT and CBT are effective interventions for LBP as standalone treatments [10, 45, 46]. Furthermore; they have the potential to be combined as a secondary intervention for prevention of LBP related disability.

It is also known that higher levels of patient self-efficacy correlate with improvements in disability [47, 48]. CBT is known for its effects on improving self-efficacy [18]. The primary aim of this trial is to assess the effects of combining MMT with MoodGYM on self-efficacy and disability . This study is the first to investigate the secondary prevention of problems in a chronic LBP population using this combined intervention approach.

If this approach shows signs of being effective in reducing long-term disability, it will allow patients, practitioners and other stakeholders who don’t have access to a multidisciplinary team, access to a best practice care tool for the management and secondary prevention of chronic LBP. Furthermore, the outcomes of this trial may produce the beginnings of a tailored ICBT program that can be added to MMT for patients that are at medium risk of developing LBP related disability and pain.

## Abbreviations

LBP: low back pain
CBT: Cognitive Behavioural Therapy
MMT: Multimodal Manual Therapy
RCT: Randomised Clinical Trial
ICBT: internet-based CBT
